# Amsterdam tool for clinical medication review: development and testing of a comprehensive tool for pharmacists and general practitioners

**DOI:** 10.1186/s13104-015-1566-1

**Published:** 2015-11-04

**Authors:** Ruth Mast, Abeer Ahmad, Sacha C. Hoogenboom, Walter Cambach, Petra J. M. Elders, Giel Nijpels, Jacqueline G. Hugtenburg

**Affiliations:** Department of Clinical Pharmacology and Pharmacy and the EMGO, Institute for Health and Care Research, VU University Medical Center, Van Der Boechorststraat 7, 1081 BT Amsterdam, The Netherlands; Department of General Practice and Elderly Care Medicine and the EMGO, Institute for Health and Care Research, VU University Medical Center, Amsterdam, The Netherlands; Institute for Rational Drug Use, Utrecht, The Netherlands

**Keywords:** Drug-related problems, Potentially inappropriate medicine, Medication review, Tool, Community pharmacy, General practice, Patients experience

## Abstract

**Background:**

Drug-related problems are prevalent among older patients, and substantially increase the risk of morbidity, (re-)hospitalisation and mortality. To detect drug-related problems and optimize treatment primary caregivers should periodically review the medication of older patients. The aim was to develop a structured, comprehensive but practicable tool to facilitate and support the reviewing of medication of older patients with a chronic disease by pharmacists and general practitioners.

**Methods:**

A tool facilitating clinical medication review by community pharmacists was developed on the basis of treatment guidelines, literature data on drug-related problems. For the identification of drug-related problems from the patient’s perspective, a script for structured interviews was developed. The tool was optimized by means of a Delphi method with an expert panel and testing in a trial.

**Results:**

The medication review tool consists of a comprehensive checklist of 124 drug-related problems divided by 20 sections according to physiological systems and diseases, and includes a structured interview script for a patient interviews.

**Conclusion:**

A structured, comprehensive and practical tool to assist pharmacists and general practitioners to perform clinical medication review including a list of potential drug-related problems in older patients with chronic disease, as well as a script for structured patient interviews, was developed.

**Electronic supplementary material:**

The online version of this article (doi:10.1186/s13104-015-1566-1) contains supplementary material, which is available to authorized users.

## Background

Drug-related problems (DRPs) are events or circumstances related to drug therapy that actually or potentially interfere with desired health outcomes [1–3]. DRPs are prevalent among older patients and substantially increase the risk of morbidity, (re-) hospitalization and mortality [4, 5]. DRPs include ineffectiveness of treatment, occurrence of adverse reactions and dissatisfaction of patients with their therapies [3]. DRPs may be the result of a wide variety of causes including medication errors, frequent medication changes, specific drug effects and drug combinations, inappropriate use of medicines, inappropriately prescribed medicines, and non-adherence to treatment [3, 6]. Factors increasing the risk of DRPs are advanced age, comorbidity, polypharmacy and a lack of coordination between different caregivers after having initiated, altered or discontinued treatments [6]. Over the years, substantial effort has been made to prevent and detect potentially inappropriate medicines (PIM) [6, 7]. Several sets of explicit criteria, of which the Beers criteria are the oldest and best known, have been developed to assist caregivers in making appropriate drug choices or assessing the quality of medication [6–8]. Explicit criteria, occasionally combined with other measures, are also used as tools to conduct medication reviews [9]. Since their introduction in 1991, the Beers criteria, and subsequently adapted sets (STOPP/START criteria) in various countries have been revised and refined with respect to structure and comprehensiveness [7, 10]. In order to detect DRPs and optimize treatment, primary caregivers should periodically review the medication of older patients with chronic diseases [6, 11–14]. Being the most comprehensive form of medication review [15], a clinical medication review (CMR) is a structured, critical examination of a patient’s medications. Its objective is to reach an agreement with the patient about treatment, optimizing the impact of medicines, minimizing the number of DRPs and reducing waste [12–16].

Although a review of medication records solely on the basis of explicit criteria may be useful, the result in terms of detected DRPs will be of limited value since medical status, clinical parameters and the way patients experience their treatment have not been considered [11, 13, 15, 17]. Therefore, only a medication review with direct input from the patient, addressing perceptions of convenience and effectiveness of treatment and eventual discomfort due to adverse events. In this way, treatment can be continued in an effective, satisfactory and safe manner [11, 15, 17, 18]. This more clinical approach to the medication review, however, requires expert judgement and is likely to be time consuming [6, 13]. Due to its comprehensive nature, the large input of data and involvement of the general practitioner (GP), pharmacist and patient, the review process must be highly structured in order to be both cost-effective and practicable [11, 13]. The availability of a tool to be used for the gathering of medication data and DRPs and their evaluation within the framework of a CMR process essentially similar to that described and used by Lowe and colleagues, would be very helpful in implementing CMR in daily practice [12, 15]. The aim of the present study was therefore to develop a structured, comprehensive, but practicable tool to facilitate and support the periodic review of older patients’ medication by community pharmacists and GPs. The tool accounts for the perspective of the patient.

## Methods

The present medication review tool is focused on the most commonly occurring chronic diseases in older persons, 65 years or older, in the Netherlands as listed by the National Institute for Public Health and Environment [19] and the medicines most frequently used to treat these disorders as listed each year by the Dutch Foundation form Pharmaceutical Statistics [20].

In the Netherlands, in primary care older patients with a chronic disease are generally treated according to the recommendations of the Dutch GP treatment guidelines [21].

With respect to criteria for appropriate prescribing and the design and implementation of a CMR, PubMed and the Cochrane library were searched using the following search terms: ‘development’, ‘tools’, ‘method’, ‘medication review’, ‘clinical medication review’, ‘drug-related problems’, ‘inappropriate medicines’, ‘inappropriate prescribing’, ‘explicit criteria’, ‘adverse effect’, ‘side effect’, ‘drug–drug interaction’ and ‘older patients’. Only full-text papers in English and Dutch up to December 2012 were included. Reference lists of identified research papers were also checked.

On the basis of the references and literature data and their clinical experience two authors in their capacity as community-pharmacist/clinical pharmacologist (JGH) and clinical pharmacologist/GP (PE) compiled a longlist of frequently occurring DRPs in older patients with a chronic disease. Medication recommended by the Dutch GP (NHG) treatment guidelines was considered appropriate, deviations were considered inappropriate. DRPs definitions were extracted from the PCNE DRP classification document (version 6.2) [3]. The PCNE classification and the list of patient-related DRPs compiled by De Smet et al. [11] were used to formulate a number of questions related to patient perceptions of drug treatment. Questions were organized in the form of a script for a structured patient interview. Data gathered during the interviews should be used to complete the DRP checklist. Results of studies on the occurrence of DRPs in older patients were used to check the DRP checklist [5, 22–25].

The CMR tool consisting of a DRP checklist and interview script was optimized stepwise by means of a Delphi procedure of two rounds with a panel of eleven experts in the field and actual testing. The experts in the field included (clinical) pharmacists, clinical pharmacologists, geriatricians and GPs with cardiovascular, asthma/COPD, diabetes or osteoporosis expertise. The panel was asked to comment on the longlist and interview script. Panel members were invited to add, change or delete items if they felt it necessary. All changes proposed were discussed by the authors. If the majority thought a change would be useful, the change was made definitive.

The CMR tool was used in a randomized controlled trial to evaluate the effect of a CMR and patient counselling on DRPs and compliance after hospital discharge [26, 27]. Using medication records, medical and patient data, the tool was manually applied in this study. On the basis of trial experiences the tool was re-evaluated with the authors of this study and several suggestions for improvement were put forward. Subsequently, in the second Delphi round, the members of the expert panel were asked to comment on the resulting tool in a similar fashion to the first Delphi round.

## Ethics

Approval of an ethics committee is not applicable.

## Results

The CMR tool comprises a comprehensive checklist of drug and patient related DRPs and a script for structured patient interviews aimed at identifying DRPs related to patient experiences with drug treatment for chronic diseases.

### The checklist

The checklist consisted of domains. Of the ten most frequently occurring chronic disorders in Dutch older patients visual disturbances (nr. 1) and deafness (nr. 5) were excluded. Since cancer treatment is given predominantly to hospitalized patients or is hospital-directed, DRPs directly related to anti-cancer treatment were also excluded. Based on the remaining seven chronic diseases, the medicines most frequently used to treat these disorders and a number of patient-related issues related to the physiological system were included. Domains were ordered into 20 sections. DRPs cover both PIM and omissions of potentially beneficial pharmacotherapy. Where appropriate, DRPs also relate to laboratory values including glucose and HbA1c levels, blood pressure and lipoprotein levels.

The resulting tool consisted of a longlist consisting of 76 DRPs. After the first Delphi round, the list increased to 126 DRPs. Examples of DRPs that were added were: ‘use of high dose of metformin and modification of diet in renal diseases (MDRD) <30 mL/min’ and ‘no antihypertensive medication in the presence of micro-vascular complications’.

After this Delphi round the tool was used in a randomised controlled trial with 340 older patients discharged from the hospital [26].

In the second Delphi round, three DRPs were deleted from the longlist and one DRP was added. The final list consisted of 124 DRPs ordered into 11 domains and 20 sections (Additional file 1: Appendix S1).

### The interview script

The interview script consisted of five sections including 34 questions addressing items such as the perceived effectiveness of medicines, side effects, problems with the taking of medicines and problems with respect to adherence (Additional file 2: Appendix S2).

## Discussion

The present study concerns the development of a structured and comprehensive tool to facilitate and support (community) pharmacists in conducting a CMR of older patients with chronic diseases in close co-operation with the patient’s GP. The CMR tool consists of a checklist of 124 common DRPs and a script for structured patient interviews used for the identification of patient-related DRPs.

In addition to several drugs-to-avoid and criteria addressing omissions of potentially beneficial medicines, the present structured list of DRPs includes important drug–drug and drug-disease interactions, dosing issues and duplicate prescriptions. With respect to these drug and disease-related items there is overlap with existing lists of explicit criteria developed to identify PIM like the Beers list, STOPP/START and the PRISCUS criteria. However, the Amsterdam Tool differs from these instruments (with the exception of STOPP/START) by being based on DRPs rather than on drugs and diseases and by containing criteria of medicine use based on laboratory values. However, most importantly the tool addresses DRPs from the patient’s perspective. Lists of explicit criteria have not been developed as instruments to support caregivers in conducting a CMR. Nevertheless, in the Netherlands, a Multidisciplinary Guideline for Polypharmacy (MDG) has recently been developed [12]. As an elaboration of the MDG for polypharmacy, STOPP/START criteria were recently translated into Dutch and supplemented by a step-by-step procedure for the performance of a CMR under the name ‘STRIP method’ [12, 28]. The ‘Amsterdam Tool for Clinical Medication Review’ can be used as an supplement to these and other methods for medication review. Many older patients with a chronic disease require specialist treatment and their hospitalization rate is well above average [6]. However, care is predominantly provided by GPs whereas medicines are supplied by community pharmacists. In the Netherlands, most patients obtain their medicines from the same pharmacy [6, 29]. Electronic pharmacy medication records (PMR) are therefore fairly complete and usually also include data on hospital prescriptions [29].

The PMR are therefore an excellent starting point for a CMR and the data contained therein are the main substrate for checking for DRPs and the subsequent evaluation. PMR, however, must be combined with medical record data provided by the GP [12]. However, in spite of the considerable amount of expert knowledge already present, additional training with respect to (geriatric) pharmacotherapy and improvement of communication skills in order to conduct structured patient interviews may be required. In this respect studies in Finland and New Zealand suggested that health care professionals did not possess the competences to conduct a CMR [16, 30].

A strength of the screening of medication using explicit criteria in the form of the CMR tool is a process that takes relatively little time to perform. On the other hand, the patient interview required to obtain information on DRPs from the patient’s perspective, the evaluation of the medication data and patient experiences in collaboration with the GP and the subsequent transfer of information to the patient and counselling are time consuming. Time consumption is also highly variable since it depends on wide variety of patient-related factors. Any measure to make a CMR more time efficient is therefore extremely useful.

Using the structured interview script will also increase the quality of the initial interview with the patient. The present tool is therefore an important contribution in terms of enhancing the efficiency of the CMR process and makes it more applicable in practice. The Amsterdam Tool for Medication Review has been used in a randomized controlled study of the effect of a CMR on the incidence of DRPs among 340 older patients discharged from the hospital [26]. In this study, the CMR resulted in a significant reduction in DRPs among patients. In particular, the DRPs ‘no drug but clear indication’ and ‘fear for side effects’ were significantly reduced. Subgroup analyses showed that the reduction of DRPs identified with medication analysis was significantly more pronounced among patients with hypertension (p = 0.011) and heart failure (p = 0.001) [26]. The results obtained with the preliminary version of the tool indicate that the application of the tool in the CMR process was effective and considered practicable.

The tool was developed on the basis of data obtained by means of a literature search on the subject of CMR and DRPs, clinical expertise and a Delphi procedure. Experts were asked to appraise the initial tool with respect to completeness, accuracy and redundancies. A limitation of our study was the lack of validation of the CMR tool. The CMR tool was tested in a randomized controlled study, but further validation is necessary. Another limitation is the rapid change in demographic pattern of diseases, evidence-based therapies and guidelines in the different countries. Therefore periodically revising the contents of the CMR tool will be necessary. The panel of experts did not include a mental health person. Given the extent of mental health issues in the elderly, a mental health person could give useful suggestions.

## Conclusion

In conclusion, we developed a structured, comprehensive and practicable tool for pharmacists and GPs to perform CMR, including a list of potential DRPs in older patients with chronic disease and a script for structured patient interviews. Future studies should address the implementation of this tool in daily practice, particularly with respect to electronic DRP screening and improving the interaction of electronic pharmacy and GP information systems in order to enhance the efficiency at the evaluation and communication stages. In addition, the effects of using the tool in CMR processes on health outcomes and costs should be investigated.

## Additional files

10.1186/s13104-015-1566-1 Checklist of (potentially) drug related problems (DRPs) in older patients with a chronic disease.
10.1186/s13104-015-1566-1 Script for patient interview (*provides information for part ‘DRPs related to the patient’ of the checklist*.

## Abbreviations

DRPs: drug-related problems
PIM: potentially inappropriate medicines
CMR: clinical medication review
GPs: general practitioners
MDRD: modification of diet in renal disease
MDG: multidisciplinary guideline for polypharmacy
PMR: pharmacy medication records
