# Supplementary material for: Amsterdam tool for clinical medication review: development and testing of a comprehensive tool for pharmacists and general practitioners
Source: BMC Res Notes. 2015 Nov 4;8:642. doi: 10.1186/s13104-015-1566-1 (PMC4632353; doi:10.1186/s13104-015-1566-1)
Supplement: Supplementary file 1 — 10.1186/s13104-015-1566-1 Checklist of (potentially) drug related problems (DRPs) in older patients with a chronic disease. [file 13104_2015_1566_MOESM1_ESM.docx]

**Appendix S1: Checklist of (potentially) drug related problems (DRP) in older patients with a chronic disease**

Introduction

This is a checklist of (potentially) DRPs which a pharmacist in close cooperation with the

general practitioner (GP) should recognize using the medication status of the patient and the

results of a patient interview.

| **A. Cardiovascular diseases** |
| --- |
| **Hypertension**  DRP based on medication status:  1. Use of Beta - blockers in combination with or non-steroidal anti-inflammatory drugs (NSAIDs) in high dose [1,2].  2. Use of more than three antihypertensive in combination with a NSAID.  3. Use of antihypertensive drugs of which the cardiovascular outcomes are not  evidence-based (prazosin, doxazosin and methyldopa).  DRP related to medical status:  4. Antihypertensive treatment does not result in a systolic blood pressure < 140 mmHg.  5. Treatment with an antihypertensive, but without regular assessment of creatinine and  potassium blood levels in patients having renal dysfunctions and using diuretics, ACE  inhibitors or angiotensin II antagonists [3].  6. Hypertension as a result of renal insufficiency, treated with antihypertensive  medication [1].  **Angina pectoris**  DRP based on medication status:  7. No use of acetylsalicylic acid (80 mg) as secondary prevention of heart disease.  8. Use of acetylsalicylic acid in combination with NSAID and no use of a stomach protector^a^ [4].  9. Use of acetylsalicylic acid in combination with selective serotonin reuptake inhibitor(SSRI) and no use of a stomach protector^a^ [4].  10. Use of clopidogrel in combination with omeprazol or esomeprazole^b^ [5,6].  11. Use of sildenafil in combination with nitrates (before 24 hours)^c^ [7].  12. Use of too much nitro-glycerine sprays (overuse).  13. Use of short-acting nifedipin capsules [8].  DRP related to medical status:  14. Target value of 50 to 60 beats per minute under maintenance treatment not achieved.  **Cardiovascular diseases including myocardial infarction, angina pectoris, stroke,**  **transient ischemic attack (TIA), aorta aneurysm and peripheral arterial disease.**  DRP based on medication status:  15. The statin dose is too low or a statin is not prescribed (first choice is simvastatin or pravastatin 40 mg) ^d^ [3].  16. No use of acetylsalicylic acid (80 mg) or other antiplatelet drug as secondary prevention of heart disease.  DRPs related to medical status:  17. LDL > 2.5 mmol/L [9].  18. Systolic blood pressure is > 160 mmHg but antihypertensives have not been prescribed.  19. Use of amiodaron in combination with thyroid dysfunction [10].  **Atrial fibrillation**  DRP based on medication status:  20. Use of a beta blocker in combination with verapamil/diltiazem^e^.  21. Rise of the digoxin level due to an interaction with verapamil (to a lesser extent also with diltiazem) and amiodaron.  22. Coumarin derivative not used, in spite of a clear indication on the basis of the Chads criteria and absence of a contra-indication for a coumarine (frequent falls, low adherence).  23. Use of digoxin and/or verapamil and/or a beta blocker in combination with sotalol, amiodaron or a class I anti-arrhythmic.  DRP related to medical status:  24. Digoxin level not monitored.  25. Serum potassium levels not monitored.  26. Heart rate is 70 – 90 beats per minute in rest or more than 110 beats per minute during light exercise.  **Systolic heart failure**  DRP based on medication status:  27. Treatment with a diuretic absent.  28. Treatment with a renin-angiotensin system (RAS) –inhibitor absent.  29. Presence of tickling cough due to treatment with an ACE-inhibitor [3].  30. Use of verapamil [11].  31. Use of diltiazem or verapamil without concomitant use of digoxin.  32. Chronic use of NSAIDs [12].  33. Use of NSAIDs in combination with a high dose of a loop diuretic (furosemide, bumetanide) or in combination with a sodium eliminating diuretic (hydrochlorothiazide) [13].  34. Use of a thiazide without concomitant use of a loop diuretic in spite of renal function impairment (creatinine clearance < 30 ml/min).  35. Statin treatment absent in spite of diagnosed heart disease and LDL > 2.5 mmol/L [3].  36. Statin dose too low (first choice is simvastatin or pravastatin 40 mg)^d^ [3].  37. Beta blocker (carvedilol, metoprolol or bisoprolol) not used.  38. Spironolactone not used in spite of New York Heart Association (NYHA) class II or III heart failure with reasonable renal function.  39. Combined use of an ACE-inhibitor and an AT1 antagonist [14].  40. Use of diuretics for static oedema but heart failure not diagnosed.  41. Ankle oedema due to calcium channel blocker use but heart failure not diagnosed.  42. Reconsider the indication for thiazides if there is a diagnosis of gout [15].  DRP related to medical status:  43. Heart failure diagnosed but NYHA classification not applied.  44. Renal function has not been assessed in the previous calendar year.  **Anticoagulant use (use related to heart disease or stroke prevention)**  DRP based on medication status:  45. Despite application of therapeutic index range for coumarin treatment (2.0-2.5), the target value of the internationalized normalized ratio (INR) of 2.5 is not reached [16].  46. Absence of gastric acid protection in patients ≥ 70 years of age.  47. No clear indication for the use of a coumarin in combination with acetylsalicylic acid and/or clopidogrel.  48. Use of the combination of a coumarin and acetylsalicylic acid in the absence of gastric acid protection.  DRP related to medical status:  49. INR incorrectly set at a value < 2 or > 3.  50. Combination of a coumarin with a platelet aggregation inhibitor not indicated. |

| **B. Artrose and rheumatic diseases** |
| --- |
| DRP based on medication status:  51. Renal failure diagnosed.  52. Acetylsalicylic acid use in combination with a NSAID, without use of a stomach protector^a^ [4].  53. Acetylsalicylic acid use in combination with a SSRI, without use of a stomach protector ^a^ [4].  54. Use of NSAIDs without stomach protection in patients ≥ 65 years of age.  DRP related to medical status:  55. Use of NSAID, although an alternative is possible, like paracetamol. |

| **C. Type 2 Diabetes Mellitus** |
| --- |
| DRP based on medication status:  56. No use of metformin^f^ [17].  57. Use of glibenclamide and frequent occurrence of episodes of hypoglycaemia [18].  58. Use of pioglitazon use in combination with a loop diuretic^g^ [19].  59. Use of hypoglycaemic agents in combination with non-selective beta blockers (except sotalol).  60. Statin dose too low (first choice is simvastatin or pravastatin 40 mg)^d^ [3].  61. Concomitant use of medicines potentially disturbing blood glucose levels, e.g. high dosed thiazides (> 12.5 mg/day) or corticosteroids (prednisone > 7.5 mg/day) [20].  62. Use of calcium channel blocker as sole antihypertensive medication.  DRP related to medical status:  63. Fasting glucose level in venous plasma is not between 4.5 – 8 mmol/L.  64. Frequent occurrence of hypoglycaemic episodes.  65. Glycosylated haemoglobin (HbA1c) > 69 mmol/L (8.5%).  66. Systolic blood pressure > 140 mmHg but antihypertensive treatment absent.  67. No use of statin in case of low-density lipoproteins (LDL) > 2.5 mmol/L or total cholesterol (TC) > 4.5 mmol/L [17].  68. Presence of (micro-) albuminuria but treatment with an angiotensin-converting enzyme (ACE) -inhibitor (or angiotensin II receptor type 1 (AT1) -antagonist) absent [21].  69. Oedema is reported in combination with the use of pioglitazon.  70. Use of high dose of metformin and modification of diet in renal diseases (MDRD) < 30 ml/min [17].  71. No antihypertensive medication in the presence of micro-vascular complications.  72. No periodic assessment (once yearly) of renal function, HbA1c, blood pressure and urinary micro-albuminuria. |

| **D. Asthma / COPD** |
| --- |
| DRP based on medication status:  73. Daily dose of beta-sympathomimetic higher than maximum dose^h^ [22].  74. Daily dose of inhalation corticosteroid higher than maximum dose.  75. Use of a beta blocker and/or timolol eye drops in spite of severe asthma status^i^ [23].  76. Use of long acting beta -2 agonists (salmeterol) as ´if necessary´.  77. Use of inhalation corticosteroid as ´if necessary´.  DRP related to medical status:  78. Disease status according to the Chronic Respiratory Disease Questionnaire (CRDQ) unknown?  79. The clinical status of the patient is not stable, but sharply fluctuating.  80. Frequent episodes of oral corticosteroids use (possible sign of compliance problems). |

| **E. Severe pain** |
| --- |
| DRP based on medication status:  81. Use of opioids without use of laxative. |

| **F. Osteoporosis** |
| --- |
| DRP based on medication status:  82. Supplementary calcium and/or vitamin D not prescribed while indicated.  83. Use of biphosphonate or denosumab without calcium and vitamin D supplementation ^j^.  84. No use of biphosphonate and no use of it previously for a maximum period of five years.  85. Chronic use of highly-dosed (> 7.5 mg/day) prednisolone or equivalent without concomitant use of a biphosphonate or denosumab.  86. Use of etidronate ^k^ [24].  87. Biphosphonate not taken on an empty stomach.  88. Simultaneous use of a biphosphonate and a Ca-, Al-, Mg-, Fe- or Zn- containing drug.  89. Severe decrease of renal function (GFR < 30 ml/min) and no use of vitamin D.  90. Use of active vitamin D metabolites in primary care setting. |

| **G. Disorders of the central nervous system** |
| --- |
| **Depression**  DRP based on medication status:  91. Use of an incorrectly-dosed antidepressant [25].  92. Duration of treatment with an SSRI too short (< 4 weeks)^i^ [25].  93. Use of SSRI longer than six months after a single episode of depression [25].  94. Use of a tricyclic antidepressant as a first choice in depression in the presence of cardiovascular risk factors or cardiovascular disease^m^.  95. Use of a tricyclic antidepressant in spite of a history of glaucoma, orthostatic hypertension or bladder retention^m^.  96. Use of a SSRI in spite of a history of hyponatremia^n^.  DRP related to medical status:  97. Continued treatment for depression in spite of lacking indication.  98. Discontinuation of antidepressant, which leads to withdrawal symptoms [26].  **Sleep disorders**  DRP based on medication status:  99. Use of other benzodiazepines than temazepam or zolpidem.  100. Hypnotic medication is used chronically and not intermittent.  101. Benzodiazepine dosage is too high.  DRP related to medical status:  102. Chronic benzodiazepine use without indication for sedation or anxiolysis at daytime. |

| **H. Disorders of the central nervous system related to cognitive function** |
| --- |
| **Psychiatric disorders**  DRP based on medication status:  103. Use of lithium without monitoring of lithium blood level, renal function, minerals (calcium, magnesium), thyroid function.  104. Unnecessary or ineffective use of anticholinergic medication (oxybutinine, solifenacine, tolteridone and promethazine).  105. No periodic evaluation of antipsychotic drug use.  **Parkinson’s disease**  DRP based on medication status:  106. Use of other antipsychotics than clozapine and quetiapine^o^.  DRP related to a medical problem:  107. Use of clozapine without monitoring of white blood count^p^.  108. Disease symptoms related to the use of neuroleptics, SSRI or metoclopramide? |

| **I. Gastrointestinal disorders** |
| --- |
| **Stomach disorder**  DRP related to medical status:  109. Indication for treatment with an acid inhibitor no longer present but treatment continued.  **Constipation**  DRP based on medication status:  110. Unnecessary and/or prolonged use of laxatives.  111. Use of codeine as prescribed for pain or coughing complaints.  112. Use of Fe supplementation without a clear indication. |

| **J. Older patients dependent on home care or in nursing homes** |
| --- |
| DRP based on medication status:  113. Medication associated with a higher risk of fall incidents (i.e. benzodiazepines, antidepressants, antipsychotics and cardiovascular medicines).  114. Use of medication with a higher risk on decline of cognition (i.e. antipsychotics and anticholinergics). |

| **L. Other problems related to the medication status** |
| --- |
| 115. Double medication is reported.  116. The indication for the drug is unknown  117. Relevant drug interactions and contra-indications are identified based on the pharmacy/GP computer information system.  118. Medication record suggesting non-adherence. |

| **K. DRP related to the perspective of the patient (to determined on the basis of patient**  **interview)** |
| --- |
| 119. Treatment indication unknown to the patient.  120. Absence of awareness how to use medication.  121. Dissatisfaction about the medication (medication as such or effectiveness of medication).  122. No trust in or doubts about the effectiveness of the medication.  123. Patient experiences adverse drug event(s).  124. Fear for adverse drug events. |

**Explanation of DRPs**

^a^ This combination of medicines results in a high risk of bleeding in the stomach

^b^ Cyp2C19 is inhibited by omeprazol. The effectiveness of clopidogrel is thereby reduced.

^c^ Risk of hypotension, followed by cardiac arrest.

^d^ Research has shown the beneficial effects of a statin on mortality.

^e^ This combination of medicines results in a risk of hypotension, disturbance in the AV

conduction, and left ventricle insufficiency.

^f^ Metformin is first choice in the treatment of Diabetes Mellitus type 2.

^g^ This combination of medicines results in a higher risk on congestive heart failure due to.

^h^ If the patient uses more than the maximum dose, an inhalation corticosteroid should be

used.

^i^ Risk of acute bronchospasm when asthma patients are using non selective Beta

blockers.

^j^ The effectiveness of these bone sparing drugs has been shown in combination with

supplementation of calcium and vitamin D.

^k^ This combination results in a high risk of GI cancer.

^l^ The antidepressant effect becomes apparent after a period of use of two to four weeks.

^m^ Tricyclic antidepressants (TCA) in depression and cardiovascular risk factors results in

a high risk of orthostatic hypotension and other cardiovascular effects

^n^ SSRIs are associated with an increased risk of hyponatraemia.

^o^ Risk of worsen Parkinson’s symptoms due to the dopaminergic effect of antipsychotics.

^p^ Clozapine may cause a severe reduction in white blood cells (agranulocytosis).

**References**

1. Stewart PM: Mineralocorticoid hypertension. *The Lancet* 1999;353:1341-1347.

2. McGettigan P, Henry D: Cardiovascular Risk with Non-Steroidal Anti-Inflammatory

Drugs: Systematic Review of Population-Based Controlled Observational Studies. *PLoS*

*Med* 2011;8:e1001098.

3. Multidisciplinary guideline on cardiovascular risk management (revision). NHG,

Utrecht, 2011. Available from https://www.nvvc.nl/UserFiles/File/Pdf/cvrm_2006.pdf.

Accessed April 12, 2013.

4. Loke YK, Trivedi AN, Singh S: Meta-analysis: gastrointestinal bleeding due to

interaction between selective serotonin uptake inhibitors and non-steroidal antiinflammatory

drugs. *Alimentary Pharmacology & Therapeutics* 2008;27:31-40.

5. Kwok CS, Loke YK: Inconsistencies surrounding the risk of adverse outcomes with

concomitant use of clopidogrel and proton pump inhibitors. *Expert Opin Drug Saf* 2012; 11:275-284.

6. Ho P, Maddox TM, Wang L: Risk of adverse outcomes associated with concomitant use

of clopidogrel and proton pump inhibitors following acute coronary syndrome. *JAMA*

2009;301:937-944.

7. Ishikura F, Beppu S, Hamada T, et al. Effects of sildenafil citrate (Viagra) combined with

nitrate on the heart. *Circulation* 2000;102:2516-2521.

8. Jung S, Choi N, Kim J, et al. Short-acting nifedipine and risk of stroke in elderly

hypertensive patients. *Neurology* 2011;77:1229-1234.

9. Collins R, Armitage J, Parish S, et al; Heart Protection Study Collaborative Group.

MRC/BHF Heart Protection Study of cholesterol-lowering with simvastatin in 5963

people with diabetes: a randomised placebo-controlled trial. *Lancet* 2003;361(9374):2005-2016.

10. Ahmed S, Van Gelder IC, Wiesfeld ACP, et al: Determinants and outcome of

amiodarone-associated thyroid dysfunction. *Clinical Endocrinology* 2011;75:388-394.

11. Hoes AW, Voors AA, Rutten FH, et al: NHG-Standaard Hartfalen. *HUISARTS*

*WETENSCHAP* 2010;53:368-389.

12. Marcum ZA, Hanlon JT: Recognizing the Risks of Chronic Nonsteroidal Anti-

Inflammatory Drug Use in Older Adults. *Ann Longterm Care* 2010;18:24-27.

13. Heerdink ER, Leufkens HG, Herings RC, et al: Nsaids associated with increased risk of

congestive heart failure in elderly patients taking diuretics. *Archives of Internal*

*Medicine* 1998;158:1108-1112.

14. Mann JF, Schmieder RE, McQueen M, et al: Renal outcomes with telmisartan, ramipril,

or both, in people at high vascular risk (the ONTARGET study): a multicentre,

randomised, double-blind, controlled trial. *The Lancet* 2016;372:547-553.

15. Choi HK, Oriano LC, Hang Y, et al: Antihypertensive drugs and risk of incident gout

among patients with hypertension: population based case-control study. *British Medical*

*Journal* 2012; 344.

16. NHG-werkgroep Diepe veneuze trombose en longembolie: NHG-Standaard Diepe

veneuze trombose en longembolie. *HUISARTS WETENSCHAP* 2015;58:26-35.

17. Rutten GEHM, De Grauw WJC, Nijpels G, et al: NHG-Standaard Diabetes mellitus type

2. *HUISARTS WETENSCHAP* 2013;56:512-525.

18. Holstein A, Plaschke A, Egberts EH: Lower incidence of severe hypoglycaemia in

patients with type 2 diabetes treated with glimepiride versus glibenclamide. *Diabetes*

*Metab Res Rev* 2001;17:467-473.

19. Filion KB, Joseph L, Boivin JF, et al: Thiazolidinediones and the risk of incident

congestive heart failure among patients with type 2 diabetes mellitus. *Pharmacoepidem*

*Drug Safe* 2011;20:785-796.

20. Suissa S, Kezouh A, Ernst P: Inhaled Corticosteroids and the Risks of Diabetes Onset

and Progression. *The American Journal of Medicine* 2010;123:1001-1006.

21. Hughes DB, Britton ML: Angiotensin-Converting Enzyme Inhibitors or Angiotensin II

Receptor Blockers for Prevention and Treatment of Nephropathy Associated with Type

2 Diabetes Mellitus. *Pharmacotherapy: The Journal of Human Pharmacology and Drug*

*Therapy* 2005;25:1602-1620.

22. Fanta CH: Asthma. *N Engl J Med* 2009;360(10):1002-1014.

23. Salpeter SR, Ormiston TM, Salpeter EE: Cardioselective beta-blockers for reversible

airway disease. Cochrane Database of Systematic Reviews: Reviews 4, 2002.

24. Vestergaard P: Occurrence of Gastrointestinal Cancer in Users of Bisphosphonates and

Other Antiresorptive Drugs Against Osteoporosis. *Calcif Tissue Int* 2011;89:434-441.

25. Van Weel-Baumgarten EM, Van Gelderen MG, Grundmeijer HGLM, et al: NHG

Standaard Depressie M44. *HUISARTS WETENSCHAP* 2012;55:252-259.

26. Warner CH, Bobo W, Warner C, et al: Antidepressant discontinuation syndrome. *Am*

*Fam Physician* 2006;74: 449-456.
