# Supplementary material for: Amsterdam tool for clinical medication review: development and testing of a comprehensive tool for pharmacists and general practitioners
Source: BMC Res Notes. 2015 Nov 4;8:642. doi: 10.1186/s13104-015-1566-1 (PMC4632353; doi:10.1186/s13104-015-1566-1)
Supplement: Supplementary file 2 — 10.1186/s13104-015-1566-1 Script for patient interview (provides information for part ‘DRPs related to the patient’ of the checklist. [file 13104_2015_1566_MOESM2_ESM.docx]

**Appendix S2: Script for patient interview (*provides information for part ‘DRPs related to the* *patient’ of the checklist.***

*1. Preparing the interview:*

- Which chronic medicines is the patient using according to the pharmacy information system?

*2. Start of the interview:*

- Which medicines are you using?

- Are you using other medicines than prescribed by the general practitioner/specialist, like painkillers or vitamins?

- Is someone helping you with the management of your medicines?

*3. Effectiveness of the medicines:*

- Do you know for what disease or symptom you have to use your medicine?

- Is your medicine effective?

- Why do you think your medicine is effective or not?

- Do you know when to take your medicine, like before/after a meal or before sleeping?

- Do you know how to use your medicine?

*4. Adverse drug effects of the medicines:*

- Are you experience adverse drug effects of your medicines?

- Of which medicine do you experience an adverse drug event?

- Which adverse drug event do you experience?

- How long do you experience this adverse effect?

- Are you afraid to experience adverse drug effects of your medicines?

- Of which medicine are you afraid to experience adverse drug effects from?

- For which adverse drug effect you are afraid?

- Have you, once or more, been fallen in the past year?

*5. Problems with the use of the medicines:*

- Do you sometimes forget to use your medicine?

- Which medicine do you sometimes forget to use?

- What was the reason you forget to use the medicine?

- Do you have a method which remembers you to use your medicines?

- Do you sometimes consciously not use your medicines?

- Which medicine are you sometimes consciously not using?

- What is the reason you sometimes consciously not use the medicine?

- Have you ever stopped using your medicine on your own initiative?

- With which medicine you stopped on your own initiative?

- What was the reason you stopped the medicine on your own initiative?

- Is it difficult for you to take your medicines?

- Which medicine is difficult to take?

- Why is it difficult for you to take this medicine?

- Are you getting help with the use of your medicines?

- What kind of help did you get with the use of your medicines?

- Would you, if possible, use a combination drug?

- Are you generally satisfied with your medicines?

Thank you for your time.
